# Influence of Poly(Ethylene Glycol) End Groups on Poly(Ethylene Glycol)-Albumin System Properties as a Potential Degradable Tissue Scaffold

**DOI:** 10.3390/jfb10010001

**Published:** 2018-12-24

**Authors:** Robyn J. Overby, Dale S. Feldman

**Affiliations:** Department of Biomedical Engineering, University of Alabama at Birmingham, Birmingham, AL 35294, USA; robynnotes@gmail.com

**Keywords:** tissue engineering scaffold, degradable-regenerative scaffolds, functionalized polyethylene glycol, albumin, swellability

## Abstract

Chronic dermal lesions, such as pressure ulcers, are difficult to heal. Degradable tissue scaffold systems can be employed to serve as a provisional matrix for cellular ingrowth and facilitate regenerative healing during degradation. Degradable regenerative tissue scaffold matrices can be created by crosslinking albumin with functionalized poly(ethylene glycol) (PEG) polymers. The purpose of this study was to evaluate the stability of PEG-albumin scaffold systems formed using PEG polymers with three different functionalized end chemistries by quantifying in vitro system swellability to determine the most promising PEG crosslinking polymer for wound healing applications. Of the three polymers evaluated, PEG-succinimidyl glutarate (SG) exhibited consistent gelation and handling characteristics when used as the crosslinking agent with albumin. PEG-SG polymers were identified as an appropriate synthetic crosslinking moiety in a PEG-albumin scaffold system, and further in vitro and in vivo evaluation of this scaffold system is merited.

## 1. Introduction

Chronic skin wounds, such as pressure ulcers, are particularly hard to heal due to their typical lack of adequate nutrient support, mechanical load, contamination, and underlying tissue pathology [1]. Treatment methods for chronic wounds can fall short due to an overall lack or inappropriate speed of cellular ingrowth and vascularization. The medical resource, time, societal, and financial burden of these wounds is high [2]. The annual incidence of pressure ulcers in the United States is approximately 2.5 million patients with associated costs exceeding US$11 billion/year [2,3,4]. Medicare and medicaid are the primary payers in over 85% of the hospital stays associated with pressure ulcers; therefore, the taxpayer burden is also high [3]. Pressure ulcers associated with hospitalizations are increasingly common with the incidence increasing nearly 80% between 1993 and 2006 [3]. Chronic wound rates will continue to rise due to an aging population, the prevalence of medically complicating conditions like diabetes and obesity, and the volume of surgical procedures performed annually [2].

Treatment methods required for pressure ulcers are dependent on the stage of the wound and extent of tissue loss. Management of full thickness wounds typically includes either surgical or non-surgical treatment options. Non-surgical treatment methods require approximately six months of bed rest/offloading and continual dressing changes [5]. Conservative non-surgical methods of treatment are only effective in healing 50% of Stage III (full thickness tissue loss) and 30% of Stage IV (full thickness tissue loss with exposed muscle or bone) within a six-month timeframe [6]. Surgical treatments can include direct closure, skin grafts, skin flaps, musculoskeletal flaps, or free flaps [6]. Surgical management can pose additional risks due to the patient comorbidities, lack of viable tissue options, and the creation of additional wound sites in poorly healing patients. Although offering improved healing rates, surgical management also requires substantial bed rest, approximately six weeks, and has high rates of recurrence [5].

Surgical and non-surgical management of pressure ulcers are both costly. On average, a pressure ulcer increases the costs of a hospital stay by over US$40,000 [2]. Individual pressure ulcers may cost between US$20,000 and US$150,000 to treat [4]. These costs do not take into account the socioeconomic burden due to disability and loss of wages or the quality of life, emotional toll, and social impact that the longevity of these non-healing wounds may present [2]. Therefore, there is a need to develop a non-surgical treatment method for chronic wounds that offers comparable healing rates to surgical treatment methods without significant cost inflation, quality of life deficits, or utilization hurdles. The goal of this study was to evaluate a potential regenerative scaffold material to be used to speed healing rates in non-surgical treatment management of chronic wounds, such as pressure ulcers.

Applying a material directly to the wound bed to serve as a degradable regenerative scaffold system, alone or in conjunction with other treatment modalities, may be an easy, low risk, inexpensive modality to faster and more effective healing in chronic wounds. In tissue scaffold systems, a biomaterial placed in the wound bed provides a temporary structure that facilitates a more rapid movement of cells into the open space between wound edges. It is believed that this movement of cells resembles the regenerative epithelialization that occurs as superficial wounds heal [1]. Once cells have infiltrated the open space, they can break down the material found there and replace it with new tissue. Ideally, the tissue scaffold system degrades through a biofeedback-controlled mechanism. Although a multitude of tissue engineered skin substitutes and individual components (growth factors and cell therapies) are commercially available, the disadvantages and limitations of available substitutes necessitate further material investigations [7,8]. Specifically, the use of tissue-engineered skin substitutes has not proven to sufficiently reduced the healing time of non-surgical treatments to a comparable rate with surgical methods [7,9,10,11].

Scaffolds for tissue engineering contribute to the repair and regeneration of the wound by offering a suitable medium for cellular factors, cellular proliferation, and differentiation. Historically, scaffolds for skin tissue engineering have included fibrous scaffolds, acellular matrices, hydrogels, microsphere scaffolds, and composite materials [12]. Naturally derived components in a tissue scaffold system resemble the native extracellular matrix, contribute to a suitable environment for cellular ingrowth, and are biocompatible and easily degraded through normal biological processes. Synthetic biomaterials offer increased strength and tailorability to a tissue scaffold system [12]. By combining both natural and synthetic components in a tissue scaffold matrix, the resultant system can be both biologically favorable for regeneration and manipulatable for targeted material properties. When natural–synthetic composite tissue scaffold system properties are understood, the system can be modified to produce desired physical properties, like mimicking the degradation rate of a specific wound type. In this study, the stability effects of changing the chemical structure of the synthetic component of a natural–synthetic composite tissue scaffold system were evaluated since previous studies using a similar system offered slower degradation profiles that did not sufficiently match the healing rate in an open wound and could not maximize the scaffold benefit [13].

Examples of natural materials that can be incorporated into a matrix to facilitate biofeedback control include fibrin, collagen, and albumin [14,15,16,17,18]. Each of these materials can be crosslinked to form a provisional matrix through either biological or synthetic means [14,15,18]. Often, they are used in conjunction with other techniques such as skin grafts or artificial skin applications. When combined with other treatments, natural scaffolds are used to adhere the grafts to the wound site as well as provide a provisional matrix to facilitate cell movement into the area. Given that natural scaffolds conform to the wound bed, their use allows for more complex contours to be treated through grafting or skin substitutes. The degradation of a natural scaffold is controlled through the regeneration of the wounded area. This biofeedback-controlled mechanism is driven by material degradation through enzymatic and phagocytic means governed by the cells rather than the independent hydrolytic degradation of synthetic materials [19]. The hope is that a scaffold effect is created via a degradation rate comparable to or a little slower than the ingrowth rate for natural scaffolds, but in many cases, they are mismatched [10]. The addition of a synthetic component to the natural component can allow fine tuning of the degradation rate.

Although all three biological materials have benefits [15,16,17], albumin was chosen for its successful medical history, physiological properties, relative ease of processing, availability, reduced expense, and ability to incorporate a synthetic component. Albumin is the most abundant serum protein, has a flexible loops structure that can change in a multitude of environments, and can withstand greater temperatures than fibrin during viral inactivation. Compositionally, albumin contains large quantities of the amino acids cysteine, leucine, glutamic acid, and lysine. The overall ionic nature of the protein contributes to the solubility and hydrophilic characteristics expressed. It denatures when applied along a surface or when exposed to extreme pH conditions, high temperatures, or various solvents. Albumin does not unfold until exposed to pH values less than 3 or greater than 10. It does, however, undergo isomerization in response to changes in pH. Albumin is degraded by endocytosis eventually leading to lysosomal breakdown. Its degradation is more effective if the albumin is modified or denatured since macrophages search out such alterations. This is particularly true of modifications located at ε-amino acids (i.e., lysine). Metabolism of albumin results in free amino acids that may be used for the biosynthesis of other products. The skin naturally functions as a primary site of extracellular albumin storage and metabolism [20].

Albumin, unlike fibrin and collagen, is not crosslinked in its native form. Poly(ethylene glycol) (PEG) has been used previously to crosslink albumin as well as other proteins [13,21,22,23]. PEG is a neutral, flexible, adaptable, nontoxic, water-soluble polymer with the basic structure: HO–(CH_2_CH_2_O)*_n_*–CH_2_–CH_2_–OH.

The structure of PEG can be altered by varying the molecular weight of the molecule, modifying the PEG ends for binding purposes, or changing the shape through branching techniques. In an aqueous solution, PEG molecules have a large hydrodynamic radius due to their hydrophilic nature [24]. In fact, studies indicate that an average of two to three water molecules are bound per repeating PEG unit [25]. The trihydrated structure of the ether groups on the PEG backbone occurs when chain molecular weights exceed 600 [26]. In separation techniques, this property makes PEG look larger than other molecules of equivalent molecular weight. In vivo this can help retain substances quickly cleared from the body. Since PEG is soluble in both aqueous and organic solvents, it can interact with both the intra- and extra-cellular spaces as well as the cellular membrane biologically. In aqueous PEG solutions, these positive interactions with cells provide an environment suitable for cellular infiltration and growth [24].

**Figure 1:**
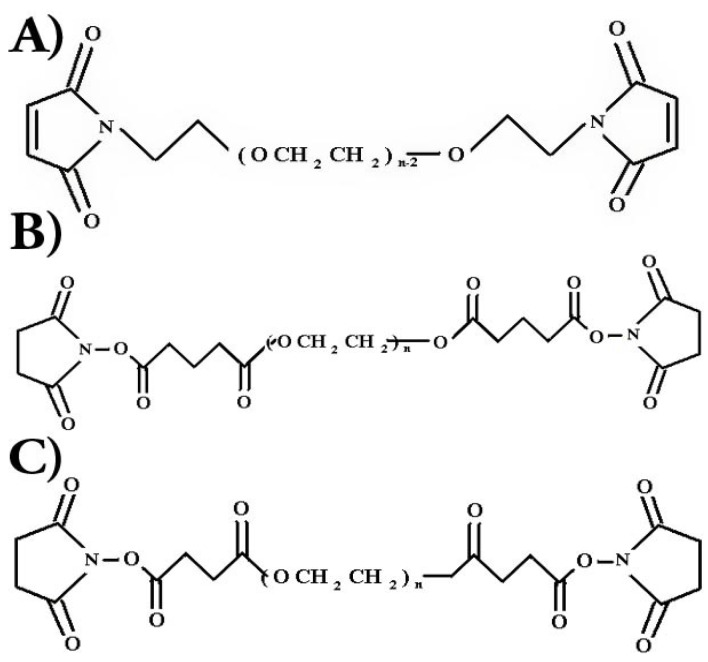
Chemical structures of (**A**) PEG-MAL_2_, (**B**) PEG-SG_2_, and (**C**) PEG-SS_2_.

PEG polymers can be modified to create more specifically activated ends. Examples of such endings used to activate PEG include succinimidyl propionate (SPA), succinimidyl succinate (SS), succinimidyl glutarate (SG), and maleimide (MAL). The chemical structure of these polymers can be seen in Figure 1.

These various PEG functional end groups can be used to target specific chemical compositions and induce binding with substances such as proteins. Nitrile hydroxyl succinide (NHS) esters replace the hydroxyl groups on the ends of a methoxy polyethylene glycol in each of the succinimidyl end groups named. The end esters of these groups then form amide linkages with ε amino acids such as lysine. The stability of each NHS PEG is dependent on the configuration of the PEG backbone as well as the presence of hydrolytically cleaved functional groups such as esters. Since PEG-SPA molecules do not contain esters in their backbone like PEG molecules modified by SS or SG, they tend to be more stable. End groups, such as MAL, operate by attacking thiol groups found in cysteine and methionine amino acids. PEG molecules containing multiple functional end groups can also be created to serve as a cross-linking agent between proteins [21,22]. The space between cross-links is altered by changing the molecular weight between active ends or by altering the number of functional ends in the PEG molecule. Although the polyether portion of PEG molecules do not undergo enzymatic degradation, the ester linkage between the PEG and the succinic ester end in a NHS capped PEG polymer lose stability in aqueous solutions over time. In physiologic environments, PEG chains will experience hydrolytic cleavage from activated ends. These chains can then be cleared from the body through organs such as the kidney [27].

PEG and albumin have both been used independently in medical applications for decades [20,22,24]. Together these materials combine to form a unique substance that is strong, adhesive, biodegradable, and nonimmunogenic [13,23,28]. The solid properties of this system are made possible through multiple functional end groups terminating the ends of the PEG chain. These groups can be tailored to target and covalently bind to specific chemical species in protein amino acids. These binding sites serve as crosslinks between albumin chains, creating a network, limiting mobility, and yielding a solid gel. This PEG-albumin structure has promise in a variety of applications. It, however, seems particularly well suited for initiating the healing process of a subcutaneous open wound since it can hasten cell movement into the open spaces of a wound by serving as a temporary scaffold. As the cells degrade the PEG-albumin scaffold, they will in turn replace the material with new tissue, eventually filling the entire wound. The bi-modal degradation pathway of the scaffold established through the two different mechanisms of metabolism for the synthetic and natural components of the system offer greater control of system properties. By understanding the characteristics of this material, optimized systems with different degradation rates and stability profiles can be created to effectively and efficiently treat wounds with different healing rates by adjusting the composition of the two components.

Examples of how systems parameters could be altered to optimize properties include changing the length of the PEG crosslinker by changing the molecular weight, relative quantities of proteins and PEG, the type of PEG end group utilized, or the number of end groups per PEG chain. It is believed that these changes will alter the degradation rate of the system as well as other properties in vivo. In past studies, the greatest limiting factor of the PEG-albumin scaffold breakdown process was the rate in which the system degraded in vivo [13]. In incision wounds, the scaffold broke down at a rate similar to fibrin but took significantly longer for an open wound. Wound closure results were most promising for the 30/10 albumin/PEG-SPA adhesive. In order to optimize the PEG-albumin system in vivo, it is imperative that connections between initial system composition and handling properties are understood in vitro. Assuming biofeedback degradation of albumin remains consistent per wound type, alterations in PEG polymer characteristics should offer increased control of scaffold performance by impacting hydrolytic degradation of the scaffold. Swellability studies were chosen as the means of evaluation in the initial identification of PEG type for a wound healing system since the hydrogel’s ability to swell could potentially affect the surface area in contact with the wound, permeability of the scaffold to solutes and cells, and release kinetics of the network. The swellability of the crosslinked network in specific environments is innately linked to the types of components used, quantities of those components, and the resultant crosslink density of the system. A system’s ability to swell offers insight into the material strength, degradation, stability, and space available for expansion between network links. In a previous study, in a similar system changes in swellability due to crosslink length, crosslink density, and pH resulted in similar changes in hydrolytic degradation rates [10,19]. The purpose of this study was to investigate system stability of three functionalized PEG types, PEG-MAL_2_, PEG-SG_2_, and PEG-SS_2_, used as the crosslinking moiety in a PEG-albumin scaffold through an in vitro swellability study. Since dermal wounds exhibit dynamic environments over time, swellability studies were performed in two different pH environments. It is hypothesized that changing the chemistry of the PEG functional end groups will alter the system stability. By investigating the correlations between the PEG structure and their functional outcome, appropriate PEG-albumin systems can be chosen for further in vitro and in vivo investigations.

## 2. Results

Based on test results provided by the supplier, the properties of the PEG used can be seen in Table 1.

During swellability tests, PEG-MAL_2_ samples would not cure under any circumstances over any length of time. PEG solutions were altered to pH levels of 5.0, 6.0, 7.0, and 8.0 to create an environmental conducive to gelation. In many cases, the PEG-SS_2_ specimens were deemed inadequate due to their inability to cure. The PEG-SS_2_ samples that would form gels, however, required cure times often exceeding four hours. Specimens made with PEG-SG_2_ formed gels within reasonable time periods in all cases.

**Figure 2:**
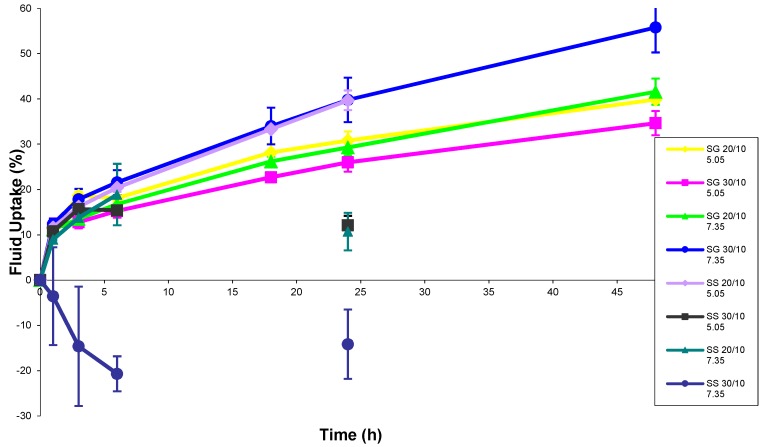
Short term fluid uptake of PEG-SG_2_-albumin and PEG-SS_2_-albumin scaffolds as a percentage over time with sample standard deviations. Samples are classified by PEG functional end group (succinimidyl glutarate (SG) or succinimidyl succinate (SS)) and albumin:PEG ratio (20/10 or 30/10) gel system properties and PBS solution pH (5.05 or 7.35) used for testing.

**Figure 3:**
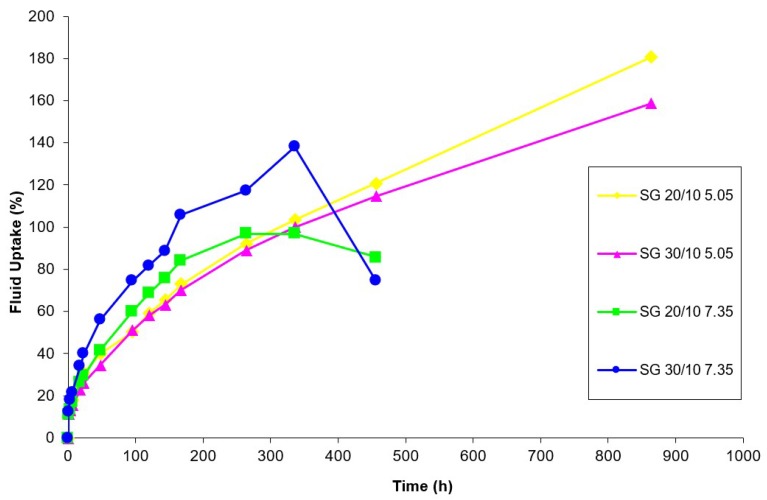
Long term fluid uptake and swellability of PEG-SG_2_-albumin scaffolds as a percentage of fluid uptake over time. Samples were classified using the albumin:PEG ratio (20/10 or 30/10) gel system properties and PBS solution pH (5.05 or 7.35) used for testing.

Samples were allowed to swell until they did not exhibit the properties of a solid and behaved like a liquid. During testing, the nature of the specimen was assessed during the decanting process. Any portion of the system that could not be distinguished from the solvent because it did not adhere to a portion of gel mass during solvent removal was also discarded. In general, system swelling tended to follow the same pattern. Systems would swell continuously over time until they reached an undefined threshold of fluid uptake. After reaching their swelling threshold, systems would lose gel properties quickly to become a viscous liquid. Short term rates of fluid uptake are depicted in Figure 2, while long term percentages of fluid uptake are depicted in Figure 3. Table 2 offers sample properties at key timepoints. The PEG-SS_2_ specimen exhibited swelling properties within the first 24 h of testing. No PEG-SS_2_ specimen exhibited gel properties at evaluation timepoints past 24 h. PEG-SS_2_ samples had limited stability in the short term and did not exhibit long-term stability in this model. Differences among systems with PEG-SG_2_ and PEG-SS_2_ functional end groups over time were statistically significant using ANOVA (analysis of variance) (*p* < 0.05). Based on the PEG-SG_2_ system gelling consistency and the stability of the gel in an aqueous environment over time, the properties of a PEG-SG_2_ -albumin scaffold should be investigated in further studies.

## 3. Discussion

Although the results from functional end chemistry swellability tests did correlate well with general hypotheses that changing the functional end group would alter scaffold stability, the extreme differences among PEG-MAL_2_, PEG-SS_2_, and PEG-SG_2_ systems were surprising. The PEG-MAL_2_ system was chosen to explore the possibility of crosslinking the albumin molecule at amino acids containing sulfhydryl functional groups rather than amine functional groups. Although these links were more specific, this type of connection was considered since a more targeted crosslink point would improve the short-range order of the system by creating a more homogeneous and predictable structure when components were mixed thoroughly. Improved homogeneity of the scaffold structure could have an effect on the degradation of the system and release characteristics of any bound therapeutics such as growth factors. Unfortunately, the PEG-MAL scaffold systems would not gel. Curing problems were first attributed to the pH of the N-2-Hydroxyethylpiperazine-N’-2-ethanesulfonic acid (HEPES) buffer system since both Roberts and Veronese reported higher activity in milder or neutral conditions that collectively ranged between 6 and 8.5 pH [29,30]. This system’s inability to gel, however, was not dependent on the pH of the HEPES buffer used to solvate the PEG since the PEG-MAL_2_-albumin system still did not cure in any circumstances after any period of time when the pH was lowered to levels of 5.0, 6.0, 7.0, and 8.0 in an effort to increase activity. The system’s lack of cure may have been due to the low activity of the starting PEG-MAL_2_ material. As seen in Table 1, the functional end group activity of the PEG-MAL_2_ was close to 40% lower than the activity levels reported for the other PEG functional end chemistries. This low activity may be directly related to the processing difficulties experienced when manufacturing this functionalized PEG. The PEG-MAL_2_ gelation problem may be inherent to the amino acids targeted within the albumin sequence. Since PEG-MAL_2_ targets amino acids with sulfhydryl functional groups, there are only two qualifying amino acids, methionine and cysteine. Although cysteine residues occur 35 times within the albumin sequence, only one of these residues was not involved in forming a disulfide bond. Methionine also occurred four times within the sequence; however, the accessibility of these specific amino acids may not be adequate in the normal albumin conformation. It is believed that this system could be of more benefit if used to bind specific antibiotics or factors to an albumin scaffold crosslinked via other PEG types. In the future, this may be a helpful drug delivery doping mechanism for molecules with a naturally or chemically engineered high number of sulfhydryl groups. As the means for forming crosslinks among albumin molecules to create a gel, PEG-MAL_2_ would hold greater promise if the protein were denatured prior to contact with the polymer.

The most unexpected results from this PEG functional end chemistry study related to the erratic curing characteristics of the PEG-SS_2_ system and the resultant scaffolds instability in aqueous environments over time. This functionalized end group was investigated since succinimidyl succinates contained an ester bond in the backbone that was susceptible to hydrolytic degradation. Since previous studies found that systems containing PEG-SPA degraded too slowly in an open wound model [13], this functionalized end group was chosen because literature reported a more reduced stability than other succinimidyl groups [27,31]. Even though system vulnerability was expected over time, the speed of degradation was faster than anticipated. Although the presence of this ester bond does account for the short stability of the gelled systems during swellability studies, it does not explain the inconsistencies among scaffold curing capabilities. Again, such inconsistencies were first attributed to the pH levels of the HEPES buffer since Zalipsky reported that PEG-SS expressed high reactivity below a pH of 8 and deemed the polymer unstable above such values [27]. The only PEG-SS_2_ samples that gelled, however, cured at the 8.0 and 9.2 pH levels rather than the 5.0, 6.0, and 7.0 pH levels. The differences between how these systems performed and that reported in the literature may be related to the difunctional nature of the PEG-SS_2_ system examined or different interactions with the solubilizing solutions. Since the HEPES’ buffer pH was ruled out as the source of curing inconsistencies, it is hypothesized that PEG-SS_2_ was extremely sensitive to changes in environment like humidity and temperature; however, these sources of variation do not account for differences among specimens created under the same conditions. Further studies to determine the effects of environment on the PEG-SS_2_ system would be necessary to conclusively understand how gelation is affected by temperature and humidity.

The differences between the swellability performance of the PEG-SS_2_ and PEG-SG_2_ scaffolds was unexpected since time of stability differed by an order of magnitude. Zalipsky indicated that PEG-SG_2_ polymers resisted hydrolysis of the ester linkage more efficiently than PEG-SS_2_ polymers through the addition of the methyl group in the functional end group backbone [27]. This resistance was certainly apparent in our findings, as seen in Figure 3. The PEG-SG_2_ was chosen for this study since Bentz found that PEG-SG_2_ was comparable to PEG-SPA_2_ when attaching a trans-forming growth factor β2 to fibrillar collagen and delivering the growth factor in vivo [32]. This type of activated PEG has also provided success when combined with collagen and thiol activated PEG polymers [33]. In this swellability model, the PEG-SG_2_-albumin systems were able to consistently gel and maintain their structure over time. This indicates that there was sufficient network connectivity to create and maintain a hydrogel structure as well as enough space between crosslinks to allow for expansion and fluid uptake. Additional variations in molecular weight, constant functional end group quantities, and albumin:PEG ratios will offer additional insight into the tailorability of the system. Since it exhibited stable properties over time in swellability studies and has been effectively used in other research, a PEG-albumin structure utilizing PEG-SG_2_ is an appropriate and viable scaffold system to investigate as a tunable substitute for previously investigated PEG-SPA-albumin tissue scaffold systems.

Although the degradation rate of the different systems in vivo was not studied, based on previous studies, there is a direct correlation between swellability and hydrolytic degradation for these systems [10,19]. This study would provide help in selection of systems to test for applications with desired degradation rates. The ultimate goal is to develop a model to predict in vivo performance properties (including degradation rate) based on processing parameters such as chemistry and molecular weight of the PEG as well as its ratio to albumin.

To take full advantage of the tunability of these systems, however, the mechanisms for crosslinking, swelling, and degradation need to be examined. This study only considered the swellability without characterizing the chemical and structural changes among the different systems initially or over time. This would both help understand the relationships in the model as well as suggest additional modifications to control the in vivo performance.

## 4. Materials and Methods

All chemicals were purchased from Fisher Chemical (Fair Lawn, NJ, USA) unless otherwise noted. In all studies, difunctional polyethylene glycols used were purchased from Sunbio PEG-SHOP in Anyang City, Korea. Polymers were used as purchased and no further characterization was performed due to the certificate of analysis provided by the company. The three types of difunctional PEG used to investigate the effects of chemical end composition were polyethylene glycol dimaleimide (PEG-MAL_2_), polyethylene glycol-disuccinimidyl glutarate (PEG-SG_2_), and polyethylene glycol-disuccinimidyl succinate (PEG-SS_2_). Figure 1 denotes the chemical structures of these compounds. All PEG types had an average molecular weight of 10,000 ± 1000 Da. The supplier provided PEG molecular weight and polydispersity values as determined uisng GPC and MALDI-TOF methods. Functional group activity found through NMR analysis was reported as well.

Prior to use, all PEG polymers were aliquoted into 15 mL amber bottles, atmospheric air was replaced with nitrogen gas, and aliquots were stored in a dark −20 °C environment to prevent loss of end group activity. PEG solutions were prepared by solvating 0.1 g·mL^−1^ of a difunctional PEG into a basic HEPES solution with a pH 9.2 until clear. The HEPES buffer was mixed by combining 11.93 g HEPES (ICN Biomedicals, Aurora, OH, USA), 7.03 g NaCl, 0.38 g KCl, (Panvera Corp., Madison, WI, USA), and 0.432 g MgCl_2_.6H_2_O (Panvera Corp.) with distilled water in a 1000 mL volumetric flask and altering the pH with HCl and NaOH [13]. If the specimen presented an inability to gel when combined with the albumin solution, PEG solutions were prepared with alternative pH values such as 5.0, 6.0, 7.0, and 8.0

Albumin solutions were prepared by gently dissolving either 0.22 or 0.33 g·mL^−1^ of lyophilized fraction V rabbit albumin (ICN Biomedicals, Inc., Aurora, OH, USA) into a 0.85% percent sodium chloride solution until clear. Calculations were made assuming that ten percent of the lyophilized albumin was water [20]. Albumin solutions were created within twenty-four h of use, placed in amber colored bottles to prevent denaturization, and stored in a 4 °C environment until needed.

Albumin:PEG gels were formed by combining equal amounts of an albumin solution and a PEG solution with a dual syringe system (Surgical Sealant Applicator, Micromedics, Inc., Eagan, MN, USA). The syringe system was employed to ensure sufficient gel cross-linking by the simultaneous administration of equal amounts of solution. A number of different gel compositions were studied since each 0.10 g·mL^−1^ PEG type was combined with both the 0.3 and 0.2 g·mL^−1^ albumin solutions. These relative amounts of albumin to PEG are respectively denoted as 30/10 and 20/10 albumin:PEG ratios.

Specimens were formed by combining 0.25 mL of both the desired PEG and albumin solutions into a pre-weighed 1.5 mL siliconized conical centrifuge tube. Samples were immediately vortexed (Vortex-Genie2, Scientific Industries Inc., Bohemia, NY, USA) for ≈10 s to ensure homogeneous mixing. Samples were allowed to cure prior to their initial weighing. After initial weights were taken on an analytical balance (H35AR, Mettler Instruments Corp., Hightstown, NJ, USA), 1.0 mL of phosphate buffered saline (PBS) solution was placed in each conical tube. The PBS solutions had a pH of either 5.05 or 7.35. All PBS solutions were made by combining 9.88 g of biotech grade PBS powder concentrate and distilled water to create a 1 L solution. Solution pH was then adjusted using HCL and NaOH. Samples were then placed in a 37 °C controlled environment (4100 Napco Controlled Environment incubator, National Appliance Company, Portland, OR, USA). At each specified time period, the specimen PBS solution was removed, specimen weights were taken, and no more than 1.0 mL of fresh PBS was administered. Measurements were taken six times within the first 24 h, five additional times within the first eight days, and at days 11, 14, 19, and 36. Samples contained three gelled specimens (*n* = 3) at the onset of testing. Samples were classified according to functional end group type, albumin:PEG ratio, and PBS solution pH used for testing. For example, PEG-SG 20/10 5.05 specimen were formed using PEG-SG_2_ polymers with an albumin:PEG ratio of 20/10 and tested in 5.05 pH PBS solution conditions. In this study, samples tested were as follows: PEG-SG 20/10 5.05, PEG-SG 20/10 7.35, PEG-SG 30/10 5.05, PEG-SG 30/10 7.35, PEG-SS 20/10 5.05, PEG-SS 20/10 7.35, PEG-SS 30/10 5.05, PEG-SS 30/10 7.35, PEG-MAL 20/10, and PEG-MAL 30/10. Fluid uptake was determined from the difference of the specimen weight at a given timepoint from the initial specimen gel weight divided by the initial gel weight expressed as a percentage.

Statistical analysis was conducted using three-way analysis of variance (ANOVA) methods with a probability value (*p*) of 0.05 and a confidence interval of 95%. All tests were performed with Sigmastat software (Version 2.0, SPSS Inc., Chicago, IL, USA).

## 5. Conclusions

The aim of this study was to choose a suitable PEG functional end group chemistry as the crosslinking moiety for an albumin system. Of the three functional ends groups investigated in this model, PEG-SG_2_ clearly possessed superior properties since it cured consistently, cured within a practical time frame, and remained stable in an aqueous environment during swellability experiments for a reasonable period of time. Further assessment of the in vitro properties of this degradable PEG-SG_2_-albumin scaffold is merited. Specifically, the effect of PEG molecular weight, functional end ratios, and environment on matrix properties and degradation would be of benefit in determining the tailorability of the matrix for specific would healing applications.

## Figures and Tables

**Table 1:** PEG Properties.

Type	PEG-MAL_2_	PEG-SG_2_	PEG-SS_2_
**MW**	10,409	10,409	10,409
**Polydispersity**	1.045	1.045	1.045
**Functional Group Activity**	60%	98.50%	99.50%

**Table 2:** Fluid uptake (%) of PEG-SG_2_-albumin and PEG-SS_2_-albumin system data at short- and long-term timepoints.

PEG	Albumi: PEG Ratio	pH	1		3		6		24		48		96		120		144	
			Mean (%)	SD	Mean (%)	SD	Mean (%)	SD	Mean (%)	SD	Mean (%)	SD	Mean (%)	SD	Mean (%)	SD	Mean (%)	SD
PEG-SG_2_	20/10	5.05	11.39	0.77	18.61	1.62	18.12	0.86	30.79	3.48	39.87	1.86	50.39	8.03	59.17	9.43	65.44	9.20
		7.35	11.16	3.68	13.52	2.34	16.77	2.44	29.28	3.56	41.57	4.62	60.05	4.76	68.51	7.03	75.63	6.93
	30/10	5.05	11.51	3.39	12.78	0.42	15.23	0.18	26.02	3.14	34.64	5.02	50.96	6.51	58.04	6.00	63.14	7.38
		7.35	12.38	2.08	17.84	4.00	21.57	4.76	39.77	8.53	55.78	9.57	74.44	9.76	81.72	10.78	88.63	12.17
PEG-SS_2_	20/10	5.05	11.62	0.91	16.16	1.78	20.38	1.53	39.68	3.76	–	–	–	–	–	–	–	–
		7.35	8.98	0.02	13.78	0.12	18.89	0.37	10.72	3.02	–	–	–	–	–	–	–	–
	30/10	5.05	10.76	0.28	15.69	2.94	15.42	11.71	12.08	7.19	–	–	–	–	–	–	–	–
		7.35	−3.54	15.29	−14.60	18.63	−20.68	5.47	−14.14	10.83	–	–	–	–	–	–	–	–

## References

[1] Asmussen K., Sollner B. (1993). Wound Care: Principles of Wound Healing.

[2] Sen C.K., Gordillo G.M., Roy S., Kirsner R., Lambert L., Hunt T.K., Gottrup F., Gurtner G.C., Longaker M.T. (2009). Human skin wounds: A major and snowballing threat to public health and the economy. Wound Repair Regen..

[3] Russo C.A., Steiner C., Spector W. Hospitalizations related to pressure ulcers among adults 18 years and older, 2006: Statistical brief #64. http://www.hcup-us.ahrq.gov/reports/statbriefs/sb64.pdf.

[4] Haesler E., National Pressure Ulcer Advisory Panel, European Pressure Ulcer Advisory Panel, Pan Pacific Pressure Injury Alliance (2014). Prevention and Treatment of Pressure Ulcers: Clinical Practice Guideline.

[5] Maklebust J., Sieggreen M. (1996). Pressure Ulcers: Guidelines for Prevention and Nursing Management.

[6] Bluestein D., Javaheri A. (2008). Pressure ulcers: Prevention, evaluation, and management. Am. Fam. physician.

[7] Vig K., Chaudhari A., Tripathi S., Dixit S., Sahu R., Pillai S., Dennis V.A., Singh S.R. (2017). Advances in skin regeneration using tissue engineering. Int. J. Mol. Sci..

[8] Macneil S. (2007). Progress and opportunities for tissue-engineered skin. Nature.

[9] Tenenhaus M., Rennekampff H.-O. (2016). Current concepts in tissue engineering: Skin and wound. Plast. Reconstr. Surg..

[10] Feldman D. (2018). Quantification and modeling of biological processes for tissue engineering and regenerative medicine. J. Environ. Bio Res..

[11] Barrientos S., Stojadinovic O., Golinko M.S., Brem H., Tomic-Canic M. (2008). Growth factors and cytokines in wound healing. Wound Repair Regen.

[12] Chaudhari A.A., Vig K., Baganizi D.R., Sahu R., Dixit S., Dennis V., Singh S.R., Pillai S.R. (2016). Future prospects for scaffolding methods and biomaterials in skin tissue engineering: A review. Int. J. Mol. Sci..

[13] Blum B.E. (2000). Investigation of an adhesive albumin to enhance healing of full-thickness skin wounds. Ph.D. Thesis.

[14] Lou J., Stowers R., Nam S., Xia Y., Chaudhuri O. (2018). Stress relaxing hyaluronic acid-collagen hydrogels promote cell spreading, fiber remodeling, and focal adhesion formation in 3D cell culture. Biomaterials.

[15] Portnov T., Shulimzon T.R., Zilberman M. (2016). Injectable hydrogel-based scaffolds for tissue engineering applications. Rev. Chem. Eng..

[16] Spotnitz W.D., Burks S. (2012). Hemostats, sealants, and adhesives III: A new update as well as cost and regulatory considerations for components of the surgical toolbox. Transfusion.

[17] Kubinová Š. (2015). New trends in spinal cord tissue engineering. Future Neurol..

[18] Maisani M., Pezzoli D., Chassande O., Mantovani D. (2017). Cellularizing hydrogel-based scaffolds to repair bone tissue: How to create a physiologically relevant micro-environment?. J. Tissue Eng..

[19] Bowman J., Barker T., Blum B., Kilpadi D., Feldman D., Wise D.L. (2000). Tissue Adhesives for growth factor drug delivery. Biomaterials and Bioengineering Handbook.

[20] Peters T. (1996). All About Albumin: Biochemistry, Genetics, and Medical Applications.

[21] Burke S.A., Ritter-Jones M., Lee B.P., Messersmith P.B. (2007). Thermal gelation and tissue adhesion of biomimetic hydrogels. Biomed. Mater. Bristol Engl..

[22] Dozier J.K., Distefano M.D. (2015). Site-specific PEGylation of therapeutic proteins. Int. J. Mol. Sci..

[23] McCauley J., Pereboeva L., Feldman D. Wound healing treatment development for SCI. Proceedings of the Transactions of the National Injury & Violence Prevention Research Conference (SAVIR).

[24] Harris J.M., Harris J.M. (1992). Introduction to biotechnical and biomedical applications of poly(ethylene glycol). Poly(Ethylene Glycol) Chemistry. Topics in Applied Chemistry.

[25] Antonsen K.P., Hoffman A.S., Harris J.M. (1992). Water structure of PEG solutions by differential scanning calorimetry measurements. Poly(Ethylene Glycol) Chemistry. Topics in Applied Chemistry.

[26] Graham N.B., Harris J.M. (1992). Poly(ethylene glycol) gels and drug delivery. Poly(Ethylene Glycol) Chemistry. Topics in Applied Chemistry.

[27] Zalipsky S., Seltzer R., Menon-Rudolph S. (1992). Evaluation of a new reagent for covalent attachment of polyethylene glycol to proteins. BAB Biotechnol. Appl. Biochem..

[28] Sasaki H., Ohtake Y., Matsushima A., Hiroto M., Kodera Y., Inada Y. (1993). Reduction of immunoreactivity of bovine serum albumin conjugated with comb-shaped polyethylene glycol derivatives. Biochem. Biophys. Res. Commun..

[29] Roberts M., Bentley M., Harris J. (2002). Chemistry for peptide and protein PEGylation. Adv. Drug Deliv. Rev..

[30] Veronese F.M. (2001). Veronese FM Peptide and protein PEGylation: A review of problems and solutions. Biomaterials.

[31] Zalipsky S., Lee C. (1992). Use of functionalized poly(ethylene glycol)s for modification of polypeptides. Poly(Ethylene Glycol) Chemistry. Topics in Applied Chemistry.

[32] Bentz H., Schroeder J.A., Estridge T.D. (1998). Improved local delivery of TGF-β2 by binding to injectable fibrillar collagen via difunctional polyethylene glycol. J. Biomed. Mater. Res. Banner.

[33] Wallace D.G., Cruise G.M., Rhee W.M., Schroeder J.A., Prior J.J., Ju J., Maroney M., Duronio J., Ngo M.H., Estridge T. (2001). A tissue sealant based on reactive multifunctional polyethylene glycol. J. Biomed. Mater. Res. Banner.

